# Knowledge, attitude, and practice of health professionals working in emergency units towards disaster and emergency preparedness in South Gondar Zone hospitals, Ethiopia, 2020

**DOI:** 10.11604/pamj.2022.41.314.32359

**Published:** 2022-04-19

**Authors:** Sheganew Fetene Tassew, Ermias Sisay Chanie, Tekalign Amera Birle, Abraham Tsedalu Amare, Gashaw Kerebih, Tadila Dires Nega, Yeshiambaw Eshetie Ayenew, Dejen Gedamu, Gebrie Kassaw Yirga, Endalkachew Sisay Yegizaw, Dejen Getaneh Feleke

**Affiliations:** 1 Department of Emergency and Critical Care Nursing, College of Health Science, Debre Tabor University, Debre Tabor, Ethiopia,; 2 Department of Pediatrics and Child Health Nursing, College of Health Science, Debre Tabor University, Debre Tabor, Ethiopia,; 3 Department of Adult Health Nursing, College of Health Science, Debre Tabor University, Debre Tabor, Ethiopia,; 4 Department of Social and Public Health, College of Health Science, Debre Tabor University, Debre Tabor, Ethiopia,; 5 Department of Geography and Environmental Studies, College of Social Science, Debre Tabor University, Debre Tabor, Ethiopia

**Keywords:** Disaster, emergency, Ethiopia

## Abstract

**Introduction:**

catastrophe is a thoughtful community's well-being problem nowadays. Tragedies of any kind can strike at any time and have claimed many lives. Because, the emergency unit is at the frontline of disaster/emergency response system and helps as initial point to the most proper care of causalities, health professionals who are working in this area are the first caregivers, main players, and upfront role in calamity responses after pre-hospital medical services to disaster victims. The aim of this study was to assess emergency unit health professionals´ knowledge, attitude, practice, and related factors towards disasters and emergency preparedness at hospitals in the South Gondar Zone, Ethiopia, 2020.

**Methods:**

institution-based cross-sectional study with the census method was conducted at South Gondar Zone hospitals. All health professionals working in emergency units of South Gondar Zone hospitals were taken as a sample. A structured self-administered questionnaire was used to collect data. EPI-data version 4.2 and SPSS version 25 were used to enter and analyze data, respectively. The result was presented by narration, tables, and charts. Binary logistic regression was employed to determine the relations between dependent and independent variables.

**Results:**

the majority of the respondents (58.3%) were male. Regarding their profession, 52.2% were nurses, followed by physicians, 18.5%, while the rest were others. The mean age of the respondents was 29.48 ± 6.34 years. A substantial proportion (58.9%) of the study participants didn´t know whether their hospitals had a disaster management plan or not. In general, fifty-one-point seven percent´s (51.7%) of the study participants had poor knowledge toward disaster/emergency preparedness. Concerning their attitude, 55.0% had a negative attitude toward disaster preparedness. Regarding their levels of practice, 67.5% had inadequate practice disaster/emergency preparedness. Age category and profession of the respondents had a significant effect on the knowledge and attitude of respondents at P-value 0.05.

**Conclusion:**

more than half of the study participants had poor knowledge, negative attitudes, and inadequate practice about disaster/emergency preparedness.

## Introduction

Based on the World Health Organization (WHO) definition, a disaster is defined as “a sudden ecological phenomenon of sufficient magnitude to require external assistance”. It is an occasion that overcomes the resources of the region in which it occurs [1]. Disasters attack each part of the world, causing deaths and trillions of dollars in associated damage. Disasters repeatedly occur in less developed countries with poor public health organizations and insufficient health professional capabilities and usually result worsened consequential harm, considerable ecological disturbance, and ongoing psychological harm to the survivors compared to developed ones [2-4].

During a disaster, health institutions in an affected area may experience functional failure. If hospitals are affected by a major disaster, hospital roles and activities may see a noticeable reduction, and immediate external support may not always be accessible [5]. During a disaster, hospitals play an essential role within the healthcare system by providing essential medical care to their communities. Events that lead to harm to infrastructure or patient flow, such as natural or man-made disasters, often necessitate a multisectoral and multifunctional response and recovery effort, which must encompass the delivery of health care. Without proper emergency preparation, local health systems can simply become overcrowded in trying to deliver care during a critical event [6]. Disaster management sequences have four stages, those are: mitigation, preparedness, response, and recovery [7].

All actions and trials made in advance of an occurrence to ensure an active and structured response to the dangers are referred to as disaster preparedness. Disaster preparedness is critical for reducing the devastation caused by natural disasters and emergencies. Emergency preparedness can be defined as having the necessary knowledge, attitudes, and behaviors to respond to and be prepared for a disaster that has occurred or is suspected. The process of preparing and formulating policies: drill and exercise; acquiring key tools and resources required for emergency responses; and obtaining and updating the knowledge and competences of health professionals are all part of emergency preparedness [7,8]. Many public, corporate, governmental, and local institutions have begun to build disaster/emergency preparedness and response plans in order to cope with the rising weight of international pandemics and other disasters. Hospitals and health experts from those institutions are on the front lines of disaster preparedness, and they will be among the first responders in the event of a disaster [9,10].

By the end of the twentieth century, worldwide disaster preparedness thinking had resulted in identification of the critical capabilities required for a successful emergency response, as well as a greater focus on planning and practicing emergency response. The increased severity of the disease has necessitated the presence of well-organized emergency management professionals in hospitals. As a result, health professionals working in emergency rooms should be properly accomplished in the crucial information, attitude, skill, and practice required to deal with disasters. In times of chaos connected with disasters, health professionals must also be equipped to provide an effective, efficient response and assist in reducing the number of worsening outcomes [11].

Drought, floods, landslides, earthquakes, volcanic eruptions, civil instability, starvation, and disease outbreaks are all particularly common natural and man-made disasters in Ethiopia [12]. Despite the fact that disasters are widespread, limited evidence suggests that health professionals working in emergency units in Ethiopia have insufficient knowledge of disaster and emergency preparedness [13]. Despite high prevalence of disasters, there was no documented data about disaster preparedness in the study area. Therefore, the purpose of this study was to discover emergency staff health professionals' knowledge, attitudes, and practices (KAP) towards emergency and disaster preparedness at South Gondar Zone public hospital in 2020.

## Methods

**Study design:** a cross-sectional study was conducted in the emergency rooms of public hospitals in the South Gondar Zone in 2020.

**Study area:** the study was conducted at the emergency departments of South Gondar Zone public hospitals from June 1 to December 3, 2020. South Gondar is one of the zonal districts in the Amhara Region. Based on the 2007 census conducted by the Central Statistical Agency of Ethiopia (CSA), this zone has a total population of 2,051,738, an increase of 16% over the 1994 census. There are eight public hospitals in the South Gondar Zone: Debre Tabor Hospital, Addis Zemen Hospital, Ebinat Hospital, Tach Gaynt Hospital, Dr. Ambachew's Memorial Hospital, Andabet Hospital, Mekanesus Hospital, and Simada Hospital. The whole of 160 health professionals were working in the emergency units of the above hospitals during the study period.

**Source population:** all health professionals who are working in public hospitals in the Gondar Zone in the south.

**Study population:** health professionals working in the emergency departments of the public hospitals in the South Gondar Zone.

**Inclusion and exclusion criteria:** all health professionals working in emergency departments of selected hospitals were included in the study, while those who were absent due to annual leave, sick leave, or maternal leave were excluded.

### Sample size determination and sampling procedure

**Sample size determination:** the real sample size of this study was calculated by using of a single population proportion formula as follows:


$$n=\frac{{\left(Z\alpha /2\right)}^{2}*p\left(1-p\right)}{d^{2}}$$

In which n is the predicted sample size, Z is the confidence level (alpha), P is the prevalence, and d is the margin of error. The researchers used the following assumption while calculating sample size: according to a previous related study conducted at Tikur Anbesa Specialized Hospital in Addis Ababa, 50.8% professionals had good knowledge of disaster preparedness [12], 95% confidence level and 5% margin of error. The sample size was calculated to be 384 according to the above formula:


$$n=\frac{{\left(1.96\right)}^{2}*0.508\left(1-0.508\right)}{{\left(0.05\right)}^{2}}=384.06\approx 384$$

Since the target population was less than the calculated sample size using the census method, we took the whole health professional population as a sample, so the actual sample size was 160.

**Sampling procedure and data collection tools:** all health professionals working in the emergency rooms of all South Gondar Zone hospitals were interviewed using the census technique. To collect data, a standardized semi-structured questionnaire was developed with minor modifications after reviewing earlier related studies done in central Saudi Arabia emergency department health professionals, Saudi Arabia nurses, Johannesburg health care workers, and Northern Kentucky University Hospital emergency staff [14-16]. The study questioners have a whole of seventy-five questions. From those five questions were, open-ended questions, sixteen were multiple-choice questions, and fifty-four were Likert scale response statements.

**Data collection procedures:** in the time of data collection procedure, lists of emergency room health professionals were obtained from the human resource management offices of each hospital, and the whole health professionals who are working in the emergency unit were designated as a sample, and each respondent was convinced regarding the aim of the study and also, they were informed way of answering questioners. Self-administered questionnaire was disseminated to study respondents. Lastly, completeness of the data was assured by data collectors.

**Data quality assurance:** prior to actual data collection questionnaires were tested in Felege Hiwot Referral Hospital to assure quality of data. Correction of unclear and imprecise issues was conducted out based on the pre-tested results. All data collectors were rechecked for completeness of the questionnaire at the time of data collection, and thereafter investigators and supervisors undertook spot-checking and screening for completed questionnaires.

**Data processing and analysis:** before being exported to Social Science (SPSS) Version 25 for analysis, the data was coded and entered into Epi-data version 4.2. To calculate proportions, mean, median, and standard deviations, a descriptive analysis was performed. To present the processed data, simple frequency tables were employed. Regarding the knowledge levels of health professionals, the correct response was calculated by adding the whole 17 relevant multiple-choice knowledge questions for the entire of 21 multiple choices. The right choice answer was recorded by “1” and wrong choice answer was recorded as “0”. The answer was then summated from twenty-one and the median score was calculated. Consequently, the median score was ten. Lastly, individuals´ participants who scored greater than the median were considered as having good knowledge of disaster/emergency preparedness, whereas those respondents scored below median score considered as having poor knowledge of disaster/emergency preparedness.

The 17 relevant attitude questions were added together to calculate attitudes towards disaster/emergency preparedness. The responses were graded on a scale of one to five, with the options totaling 85. As a result, the median result was 55. Finally, those who scored above or equal to the median had a positive attitude toward disaster/emergency preparedness, whereas those who scored below the median had a negative attitude toward disaster/emergency preparedness. The whole set of 10 crucial multiple-choice questions, totaling 17 options, was used to assess the practice of health professionals in disaster/emergency preparedness. The correct option response was levelled as “1”, and the incorrect choice response was levelled as “0”, based on the participants' responses, and the choices were totaled out of 17. The median score was found to be three. Finally, those who scored greater than or equal to the median score were considered to have acceptable disaster/emergency preparedness, while those who scored less than the median score was considered to have inadequate disaster/emergency preparedness. Bivariant and multivariant analyses were used to determine the relationship between the dependent and independent variables. Those variables having a P value of 0.25 at a 95% confidence interval during the time of bivariate analysis were entered in to multivariate analysis to control all possible confounders.

During multivariate analysis, adjusted odds-ratios with 95 percent confidence intervals were computed to identify characteristics linked with KAP toward disaster/emergency preparedness. Independent variables with a statistically significant association with the KAP of disaster/emergency preparedness at the level of statistical significance P 0.05 and without null values in the 95% CI were reported as factors with a statistically significant association with the KAP of disaster/emergency preparedness.

### Variables the study

**Independent variables:** the independent variables of the study were socio-demographic data, year of experience, profession having disaster management plan, drills/simulation and training.

**Dependent variables:** knowledge, attitude and practice of health professional toward emergency and disaster preparedness.

## Results

**Socio-demographic characteristics of study participants:** the entire of 151 health professionals were involved in this study, giving a response rate of 94.4%. More than half of the participants (88, or 58.3%) were male and the remaining (63, or 41.7%) were female. The age of study participant was 29.48 ± 6.34 years. Concerning study participants profession majority of the study participant (79, or 52.3%) were nurses, followed by physicians, who were 28 (18.5%), and the remaining 44 (29.1%) were others (Table 1).

**Table 1:** socio-demographic distribution of health professionals working in South Gondar Zone hospitals, June, 2020

Variable	Classification	Frequency of participant	Percent (%) of participant	Mean &SD
Gender	Male	88	58.3%	
	Female	63	41.7%	
Profession	Doctor	28	18.5%	
	Nurse	79	52.3%	
	Mid wife	13	8.6%	
	Others	31	20.5%	
Highest level education	Diploma	45	29.8%	
	Degree	79	52.3%	
	Post graduate	13	8.65%	
	Specialist	14	9.3%	
Age category	20-30 years	103	68.2%	
	31-40 years	35	23.2%	29.48 ± 6.34
	>40 years	13	8.61%	
Year of experience	<1 year	85	56.3%	
	1-5 years	54	35.8%	1 ± 2.77
	>5 years	12	7.9%	

**Respondents' disaster and emergency preparedness knowledge levels:** according to the WHO definition, 53 (35.1%) of those polled correctly defined disaster, while the rest did not. The accessibility of the disaster plan in their hospital was known by 62 (41.1%) participants, while the remaining respondents were unsure whether their hospitals had a disaster preparedness plan or not. Only 19 (30.6%) of those who were aware of the availability of disaster plans knew where to find them. According to 23 (37.1%) of respondents, disaster plans are kept at the hospital's head nurse's office, emergency outpatient department team office, and disaster committee office. According to this study 95 (62.9%) of the respondents did know their specific roles through disaster drills.

Basic life support (23.2%), routine activities (7.4%), combined basic life support and routine treatment (6.3%), and 4 (4.2%) were the roles/activities stated by respondents (such as coordinating all activities, logistic supply, laboratory investigation, and delivery of a pregnant mother). According to the knowledge median score, more than half of the respondents (78, 51.7%) had inadequate disaster and emergency preparedness knowledge, while the remaining had good disaster and emergency preparedness knowledge (Table 2). The overall awareness of the emergency preparedness information questionnaire (EPIQ) among health professionals was low, according to this study; the mean score was 2.64. Seventy-two (47.7%) of the participants completed the EPIQ.

**Table 2:** knowledge towards disaster preparedness among health professional working in South Gondar Zone hospitals, 2020

Variable	Response	
	**Correct**	**Incorrect**
Define disaster	53(35.1%)	98 (64.9%)
Define disaster preparedness	46(30.5%)	105 (69.5%)
	**Yes**	**No**
Know your hospital has disaster/emergency plan	62(41.1%)	89 (58.9%)
In the last 12 months, have you seen any emergency/disaster drills	85(56.3%)	66 (43.71%)
Know alert status when emergency plan management activated	77(51%%)	74 (49%)
Know specific place for evacuation of the patient	83(55%)	68 (45%)
Are you part of emergency response team	101(67%)	50 (33%)
Know new emergency code (nickname)	0	151 (100%)
Know what emergency exercise	77(51%)	74 (49%)

**Factors associated with health professional levels of knowledge toward disaster and emergency preparedness:** based on multivariate analysis, participants between the age groups of 31-40 years were two times more knowledgeable than participants within age groups of 30 years. Nurses were twice as knowledgeable as other professionals in their field (AOR = 2.64, 95% CI, (1.06, 6.59)) (Table 3).

**Table 3:** bivariate and multivariable analysis with the level of knowledge towards disaster/emergency and preparedness and other selected variables among participants of health professional working in south Gondar Zone hospitals, 2020

Variable	Category	Levels of knowledge		AOR (95% CI)	AOR (95% CI)
		Poor	Good		
Age category	20-30 years	62	41	1	1
	31-40 years	14	21	2.22 (1.244,4.257) *	2.331 (1.04,5.20) *
	>40 years	3	10	5.56 (1.764,17.556) *	
Level of education	Diploma	26	19		
	BSC degree	21	58	0.155 (0.0.03,0.726) *	
	Postgraduate (MSC)	8	5		
	Specialist	6	8	1	
Profession	Physician	9	19	5.56 (2.62,6.73) *	
	Nurse	36	43	3.56 (1.17,4.16) *	2.64 (1.06,6.59) *
	Midwifery	8	5		
	Other	22	9	1	
Level of practice	Adequate practice	18	32	8.434 (4.61,15.44) *	5.67 (2.82,11.39) *
	Inadequate practice	60	17	1	1

**Respondents' disaster and emergency preparedness attitude levels:** unfavorable beliefs (disagree and strongly disagree) that disasters are unlikely to occur in their hospital were held by 71 (47.1%) of respondents, while 37 (24.5%) were unsure. Physicians 19 (67.9%), nurses 31 (39.2%), and midwives 6 were among the professions with the adverse view that disasters are unlikely to occur in their hospital (4%). Ninety-five (62.9 percent) of respondents did not agree (27.3 percent strongly agree and 39.1 percent strongly agree) that they would be willing to report for duty if an infectious disease outbreak occurred as a disaster at the hospital. Specialists had the greatest proportion of 5 (92.1%) of respondents, followed by nurses, who had 60 (75.9%) of respondents prepared to do so. A significant number of health workers (56, or 37.9%) refused to come to work if an infectious disease outbreak was declared a disaster.

The possible reasons for being unwilling to respond during an infectious disease outbreak were: fear of disease acquisition by self-34 (22.5%) and transmitting the disease to their family of 22 (14.6%). Based on this study, 60 (40%) of the respondents very much agreed that hospitals should be sufficiently prepared to handle all kind of tragedy, while 19 (28%), 24 (16%), 13 (8.4%) and 11 (7.4%) of the respondents agreed, were not sure, disagreed, and very much disagreed with the adequate preparation of hospitals for any type of disaster, respectively. Sixty-seven (44.4%), 47 (31.1%), 17 (11.3%), 11 (7.3%), and 9 (6%) of the respondents very much agreed, agreed, not sure, disagreed, and very much disagreed, respectively, with having a hospital disaster management plan. Regarding disaster drills, 36 (23.8%), 15 (9.9%), 60 (39.7%), 21 (13.9%), and 19 (12.6%) of the respondents very much agreed, agreed, not sure, disagreed, and very much disagreed with conducting regular disaster drills at hospital level. Generally, the study showed that 68 (45.0%) and 83 (55%) of the study participant had a positive and negative attitude towards disaster/emergency preparedness respectively.

Factors associated with levels of attitude of health professional to ward disaster/emergency preparedness. Respondents who had good knowledge were two-time more likely to have positive attitude than those with poor knowledge (AOR = 2.149, 95% CI). Regarding their experience and attitude, respondents with a work experience of five years and above were two times more likely to have a positive attitude than those who had work experience of less than five years (Table 4).

**Table 4:** factors associated with levels of attitude of health professional to ward disaster/emergency preparedness among participant of health professional working in South Gondar Zone, 2020

Variable category		Level of attitude		COR (95%CI) at p-value<0.25	AOR (95%CI) at p-value<0.05
		Negative attitude	Positive attitude		
Overall familiarity to EPIQ	Overall familiar	22	50	1.793 (1.05, 3.054) *	
	Overall unfamiliar	61	18	1	
Level of knowledge	Good knowledge	20	53	2.149 (1.26, 3.67) *	
	Poor knowledge	63	16	1	
Level of practice	Inadequate Practice	50	6	1	
	Adequate practice	33	16	1.806 (1.05, 3.09) *	
Age category	20-30 years	46	57	1	
	31-40 years	19	14	0.229 (0.067, 0.78) *	0.265 (0.074, 0.95) *
	≥40 years	3	10		1
Year of experience	Less than one years	49	36		
	1-5 years	32	22		1
	>5 years	2	10		2.0 (0.646, 4.468) *

* : statistically significant p-value<0.05; EPIQ: emergency preparedness information questionnaire

**Respondent´s level of disaster and emergency preparedness practices:** disaster drills or simulations were performed at 36 (23.8%) of the participants' hospitals. Six (4.0%) of those who responded had drilled basic life support, while 30 (19.1%) had drilled advanced traumatic life support (ATLS). Practice scores were generated to report whether the participant had successfully or erroneously answered the practice questions in order to determine the amount of practice. In addition, based on the median practice score, the scores were divided into levels. The median score for practice was 3. Higher scores indicate adequate experience, whereas lower scores indicate insufficient practice in disaster/emergency preparedness. As a result of this study, the majority of respondents (102, or 67.5%) had insufficient disaster/emergency preparedness practice.

**Factors associated with the practices of health professionals toward disaster and emergency preparedness:** based on bivariate and multivariate analysis, good knowledge and age were predictive factors for having adequate practices. Respondents who had good knowledge were two times (AOR = 2.149, 95%CI (1.26, 3.67)) more likely to have adequate practices than respondents with poor knowledge. Participants with age groups of > 40 years were 3 times (AOR = 3.49 9O5%CI (1.25, 9.68)) more likely to have adequate practices than respondents with age groups of less than 40 years.

## Discussion

In general, 48.3% health professionals in this research had good knowledge of disaster and emergency preparedness. This result was similar to that of studies conducted in southwest Ethiopia and Addis Ababa, which found 48.1% and 50.8%, respectively [12,16]. While the result was lesser than that of the study done in Saudi Arabia (65.4%) [16]. A possible reason for this difference could be the age of the studies. In Ethiopia, the era of emergency and disaster medicine is very young, while in other parts of the world it has been more than 50 years, which means that health professionals would have a different scope of knowledge about disasters.

In the current survey, 58.9% of respondents were unaware that their hospitals had a catastrophe plan in place. This result is higher than the 33 percent found in a Namibian study of a Lutheran hospital [17]. However, the conclusion of this study was significantly lower than that of a 92 percent study conducted in a Johannesburg hospital [18]. The age of participants was found to influence the level of knowledge in the current study, which revealed that health professionals over the age of 30 were approximately two times more likely than their counterparts to be knowledgeable about disaster/emergency preparedness. These findings matched those of Kenyatta Hospital in Nairobi [4]. Nurses were also twice as likely as the general public to have a better understanding of disaster and emergency preparedness. This outcome was also reported in a Kenyatta Hospital finding [4].

According to this study findings, 45% of health professional have a good attitude toward disasters and emergency preparedness. This was lower than a research done in Tikur Anbessa Specialized Hospital (TASH) found (64.8%) [12]. The probable reason for this discrepancy might be that health professionals working in TASH would have more frequent training as TASH is a specialized hospital than the current study area hospitals. Only 32.3% had adequate disaster and emergency preparedness practices, according to the findings of this survey. This result was four times higher than that of the TASH research (8.3%) [12]. The disparity could be attributable to increased contributions from various media outlets as well as an increase in unfavorable conditions, particularly during COVID-19 pandemics, which hospitals are attempting to address by delivering disaster-related drills to its health workers these days.

Furthermore, when compared to their counterparts, health professionals with adequate disaster readiness knowledge were six times more likely to exercise disaster/emergency preparedness. This result was equivalent to that of a similar study conducted at the Kenyatta National Hospital [4]. The study also showed that the educational level influences disaster/emergency preparedness practice. Specialists were 88% more likely than diploma-holder health professionals to practice disaster/emergency preparedness, and 82% more likely to have influence than degree holders. This result was supported by the discoveries of a comparable study done at the Kenyatta National Hospital [4].

**Limitation of the study:** this study used cross-sectional study which can´t show temporal relationship and it is one time study. The sample size is small even though the sapling technique was survey.

## Conclusion

Health professionals' overall knowledge of disaster and emergency preparedness was lacking. The employees had a poor level of familiarity with EPIQ in general. Around a third of the participants had gone through disaster drills and were now undergoing disaster training at the hospital. Because they were afraid of getting the sickness and transmitting it to their families, many health professionals declined to come to work during an infectious disease outbreak as a calamity.

### What is known about this topic

- Health professionals working in emergency unit has no adequate knowledge about disaster preparedness;
- Hospital emergency room (ED) professionals should be adequately trained, fitted with important knowledge, skills, and experience, and equipped to provide an effective, efficient response and aid in decreasing the number of worsen fatalities during the chaotic circumstances that accompany a disaster.

### What this study adds

- More than half of the respondents (78.7%) had inadequate disaster/emergency preparedness knowledge;
- While 68 (45.0%) of respondents had a good attitude toward disaster/emergency preparedness, 83 (55%) had a negative attitude toward disaster/emergency preparedness;
- Majority of the respondents had inadequate practice 102 (67.5%) towards disaster/emergency preparedness.
